# Syndrome of Inappropriate Antidiuretic Hormone Secretion and Trimethoprim-Related Hyponatremia Following Transurethral Bladder Wall Biopsy

**DOI:** 10.7759/cureus.17454

**Published:** 2021-08-26

**Authors:** Zahrah Nawaz, Chizukwa S Amala, Samson O Oyibo

**Affiliations:** 1 General Medicine, Peterborough City Hospital, Peterborough, GBR; 2 Emergency Medicine, Peterborough City Hospital, Peterborough, GBR; 3 Diabetes and Endocrinology, Peterborough City Hospital, Peterborough, GBR

**Keywords:** trimethoprim, syndrome of inappropriate antidiuretic hormone secretion, symptomatic hyponatremia, metabolic acidosis, hypo-osmolality, siadh, trimethoprim-related hyponatremia, transurethral bladder wall biopsy

## Abstract

Postoperative hyponatremia is common. It results from the physiological antidiuretic hormone (ADH) response to surgery and associated exacerbating factors. Common exacerbating factors include medications, excessive fluid administration, and syndrome of inappropriate antidiuretic hormone (SIADH) secretion. High-dose trimethoprim can cause transient salt-losing nephropathy, resulting in SIADH-like hyponatremia associated with hypovolemia, hyperkalemia, and metabolic acidosis. We present a patient who developed symptoms of vomiting, loss of appetite, fatigue, and abdominal discomfort six days after having a transurethral bladder wall biopsy. He had also started a course of trimethoprim two days prior to the onset of the symptoms. Initial investigations demonstrated severe hyponatremia, concentrated urine, and compensated metabolic acidosis. These results suggested postoperative SIADH possibly exacerbated by trimethoprim-related hyponatremia. Cautious IV normal sodium chloride infusion resulted in a rapid recovery. While raising the awareness of postoperative hyponatremia, this case also highlights the difficulty distinguishing between SIADH and trimethoprim-related hyponatremia.

## Introduction

Hyponatremia affects up to 30% of hospitalized patients, of which syndrome of inappropriate antidiuretic hormone secretion (SIADH) is a common cause [1]. It is likely that SIADH affects 5%-10% of hospital admissions [2]. SIADH is characterized by inappropriate secretion of the antidiuretic hormone (ADH, arginine vasopressin) despite normal or increased plasma volume. The subsequent water retention results in hyponatremia, hypo-osmolality, and high urine osmolality [2]. The causes of SIADH range from drugs, central nervous system disorders, malignancies, pulmonary disease, major surgery, and general anesthesia [3].

Symptoms are related to the degree of hyponatremia and its chronicity. A majority of patients with mild hyponatremia (serum sodium 130-135 mmol/L) are asymptomatic. Those with moderate hyponatremia (125-129 mmol/L) may present with anorexia, nausea, and malaise. Patients with severe hyponatremia (<125 mmol/L) may present with headaches, muscle cramps, irritability, drowsiness, confusion, weakness, seizures, and coma due to cerebral edema and increased intracranial pressure [2]. The diagnosis of SIADH is based on the Bartter-Schwartz criteria [4]. The criteria include hyponatremia (sodium <135 mmol/L) with corresponding hypo-osmolality (serum osmolality <275 mOsm/kg), continued renal excretion of sodium (usually >30 mmol/L), undiluted urine (urine osmolality >100 mOsm/L), absence of volume depletion, absence of other causes of hyponatremia, no recent use of diuretic agents, and the correction of hyponatremia by fluid restriction [4-6]. Treatment should be cause-specific, and fluid restriction is the first line of therapy. Increasing solute intake with urea or a combination of low-dose loop diuretics and oral sodium chloride is recommended for moderate hyponatremia. For severe symptomatic hyponatremia, a bolus dose of IV 3% hypertonic sodium chloride infusion with regular sodium monitoring is recommended [7].

Trimethoprim is a rare cause of hyponatremia. In high doses, trimethoprim has a potassium-sparing diuretic effect on the distal collecting tubules resulting in salt-losing nephropathy. Trimethoprim-related hyponatremia is usually associated with hypovolemia, hyperkalemia, and metabolic acidosis. Treatment usually includes stopping the trimethoprim and treatment with IV normal sodium chloride solution to correct the acid-base disturbance [8-9].

There are reports of patients developing SIADH after various types of major surgery and general anesthesia. Postoperative hyponatremia results in increased morbidity and hospital length of stay [10-14]. There are very few reports of SIADH following minor surgical procedures [15-17]. Trimethoprim-related hyponatremia, especially with high-dose trimethoprim or trimethoprim-sulfamethoxazole combinations, have also been reported [8-9]. We present a case of a man who developed severe hyponatremia, most likely secondary to SIADH exacerbated by trimethoprim-related hyponatremia following a transurethral bladder wall biopsy.

## Case presentation

Medical history and demographics

A 70-year-old male presented to the emergency department (ED) with a two-day history of vomiting, loss of appetite, fatigue, and abdominal discomfort.

The patient was initially under investigation for painless hematuria, for which a cystoscopy was performed. Subsequent to this (six days before presenting to ED), the patient underwent a transurethral bladder wall biopsy under general anesthesia. On the fourth day post-biopsy, the patient was also commenced on a three-day course of trimethoprim (200 mg twice a day) for suspected post-procedure infection.

Further past medical history included type 2 diabetes, for which he took metformin and simvastatin, and chronic mild anemia. His serum electrolyte levels were normal (serum sodium 133 mmol/L) prior to the surgical procedure.

On examination, the patient was orientated with a Glasgow Coma Scale of 15/15. The heart rate was 71 beats per minute, blood pressure was 164/85 mmHg, the chest was clear, and he had mild tenderness in the suprapubic region. He had no abnormal neurological signs.

Investigations

The initial venous blood results demonstrated severe hyponatremia with hypo-osmolality, and low serum bicarbonate (Table 1). The urinalysis demonstrated inappropriately concentrated urine in the presence of severe hyponatremia (Table 2). The combined results of the blood and urine analysis were suggestive of SIADH. Arterial blood gas sampling demonstrated compensated metabolic acidosis, suggesting possible trimethoprim-related hyponatremia (Table 3). Other blood results, including renal function, liver function, and thyroid function, were normal. Chest radiography and electrocardiography revealed no abnormalities.

**Table 1:** Initial blood test results.

Blood test	Result	Reference range
Sodium (mmol/L)	112	133-146
Potassium (mmol/L)	4.4	3.5-5.3
Chloride (mmol/L)	81	95-108
Creatinine (mmol/L)	97	59-104
Urea (mmol/L)	2.1	2.5-7.8
Bicarbonate (mmol/L)	17	22-29
Thyroid stimulating hormone (mU/L)	2.36	0.3-4.2
Calcium (mmol/L)	2.10	2.2-2.6
Serum osmolality (mOsm/kg)	234	275-295
Albumin (g/L)	44	35-50
9 am cortisol (nmol/L)	569	250-600
C-reactive protein (mg/L)	<10	<10
Hemoglobin (g/L)	122	130-180
White cell count (10^9^/L)	6.1	4.0-11.0
Platelet count (10^9^/L)	237	150-400

**Table 2:** Initial urine test results. A urine osmolality above 100 mOsm/kg and urine sodium concentration above 30 mmol/L in the presence of severe hyponatremia and hypo-osmolality meets the criteria for SIADH. SIADH, syndrome of inappropriate antidiuretic hormone

Spot urine test	Result
Osmolality (mOsm/kg)	558
Sodium (mmol/L)	74
Potassium (mmol/L)	43
Urea (mmol/L)	216

**Table 3:** Arterial blood gas sampling. Results indicating compensated metabolic acidosis

Arterial blood gas sampling	Result	Reference range
Arterial pH	7.42	7.35-7.45
Arterial PO_2_ (kPa)	14.03	10.67-13.3
Arterial PCO_2_ (kPa)	3.74	4.67-6.0
Arterial bicarbonate (mmol/L)	18.2	22-26
Base excess (mmol/L)	-5	-2-+2

Treatment

The patient was admitted to the intensive care unit (ICU). His oral fluid intake was restricted to 500 mL over 24 h, while he received an IV normal (0.9%) sodium chloride infusion at a rate of one liter over 24 h for two days. His serum sodium level went up to 122 mmol/L over the next 48 h, and he was stepped down to the general ward. In the general ward, the patient was continued on an oral fluid restriction of 1.5 L a day. His serum sodium continued to improve to 129 mmol/L over the next two days, and he was later discharged home.

Outcome and follow-up

A week after discharge from the hospital, a repeat serum sodium level was normal at 136 mmol/L. The bladder biopsy result indicated carcinoma in-situ and high-grade papillary bladder cancer. The patient remained well under urology follow-up.

## Discussion

Despite SIADH being common after surgery, conditions such as malignancy, pulmonary and central nervous system disorders, and medication are still more prevalent causes in hospitalized patients [2-3, 10-14]. An exhaustive review of the medication list and fluid administration is important when trying to find the cause of hyponatremia in the postoperative period.

There is a non-osmotic increase in pituitary ADH secretion during surgery, as a normal physiological response to the stress of surgery and anesthesia, resulting in mild water retention [18]. It is the presence of other exacerbating factors (e.g., medications, excessive fluid administration, SIADH) that contribute to worsening hyponatremia. SIADH can occur with any type of surgery or anesthesia. Our patient had features in keeping with stress-induced acute SIADH with possible exacerbation by trimethoprim-related hyponatremia.

Syndrome of inappropriate antidiuretic hormone is a diagnosis of exclusion [7]. Other differential diagnoses such as hypothyroidism, adrenal insufficiency, cerebral salt wasting, and occult diuretic usage need excluding. Hyponatremia due to severe hypothyroidism is usually associated with reduced cardiac output and glomerular filtration rate. Adrenal insufficiency is usually associated with hyperkalemia and orthostatic hypotension. Diuretics interfere with renal handling of electrolytes and generally contribute to diagnostic difficulties. These conditions were excluded in our patient. Another important differential diagnosis is post-transurethral resection of prostate (TURP) syndrome, which occurs after several cycles of bladder irrigation using hypotonic solutions. Bladder irrigation is performed to prevent post-surgical obstructive uropathy due to blood clot formation and retention [19]. Excess fluid is absorbed through the venous plexus/sinuses resulting in severe dilutional hyponatremia, confusion, convulsions, coma, and death. However, this patient did not have TURP or bladder irrigation. Another important differential diagnosis is dilutional hyponatremia due to excessive fluid intake. In that case, the patient’s urine would have been appropriately diluted, with an osmolality less than 100 mOsm/kg. This was not the case with our patient. Our patient also started a three-day course of trimethoprim just before the onset of his symptoms. Trimethoprim could have caused salt-losing nephropathy and generated the observed acid-base disturbance.

Distinguishing between trimethoprim-related hyponatremia and SIADH can be difficult. Trimethoprim-related hyponatremia is associated with hypo-osmolality and concentrated urine. However, there is also additional hypovolemia, hyperkalemia and metabolic acidosis, and the condition does not usually respond to fluid restriction [8-9]. Our patient was clinically euvolemic with some evidence of volume expansion, as indicated by a low serum urea level. He started low-dose trimethoprim two days before the onset of symptoms. The postoperative hyponatremia was severe at presentation, suggesting that it developed before the patient started the trimethoprim. On the other hand, our patient did have evidence of compensated metabolic acidosis and did receive two days of IV normal sodium chloride infusion. It is possible that our patient had a postoperative SIADH exacerbated by trimethoprim-related hyponatremia.

There are a number of reports of SIADH following major and minor surgical procedures and few reports of trimethoprim-related hyponatremia in the literature [8-9, 10-17]. We have reported a case of a man who underwent a surgical procedure under general anesthesia, had a course of trimethoprim, and developed features of postoperative SIADH and trimethoprim-related hyponatremia. This is one of the few reports highlighting the difficulty in distinguishing between postoperative SIADH and trimethoprim-related hyponatremia, and possibly both conditions coexisting in the same patient.

## Conclusions

Water retention secondary to increased secretion of ADH is a normal physiological response to surgery. Medications, excessive hypotonic fluid administration, and SIADH are exacerbation factors that can convert a normal physiological response to a case of severe hyponatremia in the postoperative period. This case raises awareness about postoperative hyponatremia and highlights the difficulty distinguishing between SIADH and trimethoprim-related hyponatremia.
